# A corpus-based study of modal verbs in Chinese–English governmental press conference interpreting

**DOI:** 10.3389/fpsyg.2022.1065077

**Published:** 2022-11-03

**Authors:** Yifan Zhang, Andrew K. F. Cheung

**Affiliations:** ^1^School of Foreign Languages, Huazhong University of Science and Technology, Wuhan, Hubei, China; ^2^Department of Chinese and Bilingual Studies, Hong Kong Polytechnic University, Kowloon, Hong Kong SAR, China

**Keywords:** corpus-based, modal verbs, modal values, consecutive interpreting, China

## Abstract

This study investigates the use of modal verbs in Chinese–English government press conference (GPC) interpretation. Modal verbs mark the speaker’s opinion of or attitude toward the event described in a sentence. Interpreters also use modal verbs to indicate the stances of the source language speakers. The use of modal verbs has been examined in such contexts as research papers, textbooks, and second language learners’ output; however, studies that compare differences in modal verbs between source and target languages in the context of interpreting are sparse. The investigation being reported is based on a comparable corpus—an original Chinese GPC and its English-translated version—and a parallel corpus—a translated English GPC and the original English version from the US. The results of the comparable corpus analysis indicate that the frequency of modal verbs in translated English is significantly higher than in original Chinese, in which only 40% of the modal verbs in translated English are consistent with their Chinese counterparts, while others are employed through amplification and value variation. The results of the parallel corpus analysis suggest that the increase of modal verbs in the target texts may help to achieve certain types of pragmatic functions in English.

## Introduction

Modality is the notion of intermediate degrees between positive and negative poles (Halliday, 1994), and it makes up the region of uncertainty that lies between “yes” and “no,” he also classified modalities into modalizations and modulations by the implications of the message. The use of modal verbs is an important way to encode modality. They are used in writing and speaking to express opinions, understanding, purposes, obligations, free will, and other associated notions (Leech, 1987). Modal verbs feature a complex, nonlinear mapping system between form and function due to their diverse semantic meanings (Palmer, 2014), as well as their multiple pragmatic meanings (Sinclair, 1990).

Using a corpus-based approach, this study examines the changes in modal verb frequency and value between source and target languages. It explores possible reasons for these changes from a pragmatic and semantic perspective. Through the analysis of large quantities of real language examples, corpus-based research has identified some previously neglected language structures as a complement to traditional descriptions of language systems. Researchers use large-scale corpora to observe the utilization of modal verbs with a greater degree of validity and reliability. Corpus-based studies of modal verbs tend to focus on the description of second language (L2) learners’ use of modal verbs (Westney, 1995; Palmer, 2014) and the distribution of different modal verbs employed in academic texts; however, most of these studies concern only monolingual contexts. Therefore, the question arises as to whether these verbs are used differently in bilingual settings. However, it is unusual for modal verbs to be discussed in connection to translation or interpreting, partly because modal verbs differ semantically and pragmatically from language to language. Very few interpreting studies investigate the value changes of modal verbs from ST to TT.

Since 1985, the National People’s Congress (NPC) and the Chinese People’s Political Consultative Conference (CPPCC) have been held jointly every March; these are also known as the Two Sessions. With about 2,000 to 3,000 delegates from all over China, the Two Sessions examine and approve the annual Report on the Work of the Government and make national-level political decisions. On the closing day of the NPC and the NPPCC, the sitting Chinese premier usually hosts a press conference beginning with an opening statement before opening the floor to domestic and overseas journalists for a question-and-answer session. To facilitate communication, Chinese–English consecutive interpreting is provided for both the opening statement and question-and-answer session. This gives the media access to firsthand information about the Chinese government’s attitudes and stances on important political, diplomatic, and economic issues.

Consecutive interpreting can be studied from either the interactive or textual perspective. Studies, such as those conducted by Cheung (2012a, 2014b, 2017, 2018), Pease et al. (2018), and Li et al. (2022), have investigated how different interactive and textual functions can be achieved by altering linguistic devices between source language and target language during consecutive interpreting (Cheung, 2007). Simultaneous interpreting, on the other hand, tends to be studied in terms of the cognitive aspects (Cheung, 2001, 2008, 2009b, 2012b, 2014a). Chinese officials’ statements tend to be heavily scripted. Because English is a *lingua franca* (Cheung, 2022), these Chinese statements often come with carefully crafted versions in English (Cheung, 2019; Song and Cheung, 2019; Ma and Cheung, 2020; Wu et al., 2021). The government press conferences (GPCs) on which this study focuses are no exception, as many non-Chinese-speaking correspondents may rely on prepared English versions delivered live. Studying the differences between Chinese GPCs and their corresponding English interpretations could shed light on whether the Chinese government may want it to be perceived differently between domestic and foreign audiences, as different features in the interpretation could have different impacts on listeners’ perceptions (Cheung, 2003, 2013, 2015, 2020). Using a corpus to study interpreting could illuminate both professional practice and training in interpreting (Setton, 2011).

## Literature review and theoretical framework

### Modal verbs

Huddleston and Pullum (2002) defined *modality* as the speaker’s opinion on whether a particular subject is necessary or possible. A modal verb is an auxiliary verb that implies probability, necessity, inevitability, or inclination. As their name suggests, modal verbs are important for encoding modality. The attributes of modal verbs can be summarized by the acronym NICE, which stands for *negation*, *inversion*, *code*, and *emphatic* affirmation (Huddleston, 1976, 333). Negation means that modal verbs can combine with the word *not*, so that the newly made phrase carries a negative meaning. Inversion means that modal verbs can be put in front of the subject to form an interrogative sentence. Code means that modal verbs can replace predicate verbs in sentences. Emphatic affirmation means that modal verbs can help emphasize a point.

Modal verbs differ from other auxiliary verbs in at least three ways (Palmer, 2014). First, a modal verb has the same form, regardless of whether it follows a singular or plural or a first-person, second-person, or third-person subject. Second, modal verbs do not have an infinitive or gerund form. Third, modal verbs have an exclusive relationship with one another. Thus, one should use only one modal verb at a time.

Broadly speaking, modal verbs include central modal verbs (*can*, *could*, *may*, *might*, *must*, *shall*, *should*, *will*, and *would*), marginal modal auxiliary (*dare [to]*, *need [to]*, *ought to*, and *used to*), and semi-modal verbs (*have to*, *[had] better*, *[have] got to*, *be supposed to*, and *be going to*; Quirk et al., 1985; Biber et al., 1999). Additionally, Biber et al. (1999) established a relationship between pairs of central modals according to a grammatical value based on the expression of time: *can* and *could*, *may* and *might*, *shall* and *should*, *will* and *would*.

Modal verbs are regarded as the main carriers of modal expression in many languages, including Chinese (Peng, 2007). Chinese scholars have given modal verbs various names, such as auxiliary verbs (Liu, 1960; Zheng, 2001) or modal auxiliary verbs (Tsang, 1981). However, these names and their corresponding definitions fail to capture the full range of modality between the positive and negative poles. This full range of modality is reflected in Zhu (1996) definition of *qingtaidongci* (modal verb) with respect to the semantic and stylistic characteristics of Chinese modality. Therefore, in this paper, *modal verb* also refers to Chinese auxiliaries expressing modality.

### Classification and value of modal verbs

The semantics of modal verbs has been the focus of much corpus-based research over the past decade. There are three major ways to classify modal verbs. The bipartite classification (Lyons, 1977) into epistemic and deontic modality seems to be the earliest one. Palmer (2014) further developed the bipartite model into a tripartite classification that includes deontic, dynamic, and epistemic modality. Biber et al. (1999) and Quirk et al. (1985) distinguish between intrinsic and extrinsic modality.

Among all three modal verb classifications, Palmer’s is the simplest to operationalize since it considers both semantic meaning and pragmatic functions. Thus, this paper will adapt Palmer’s classification of modal verbs into three categories: epistemic, deontic, and dynamic.

Epistemic modal verbs are used to express how certain the speaker is about a statement. For example, “what she stated *can/might/should/will/may/must* be wrong.” Deontic modal verbs are used to express how much responsibility the subject assigns to the object. For example, “you *should* get the work done by tomorrow” or “I *must* submit the paper on time.” Dynamic modal verbs can be used to express the ability or willingness of the subject. For example, “he *can* take this job” or “I *will* join this team on the project.”

As each modality has a certain magnitude and pragmatic orientation (Halliday, 1994), the speaker can alter the pragmatic orientation of the discourse based on the value implied in the modality expression. Thus, the affirmative and negative of one modal verb might have different values. Halliday (1994) believes that the modality of positive modal verbs (modal operators) can be roughly divided into three levels based on their modal intensity: low, medium, and high. In terms of pragmatic orientation, the lower the value of modal verbs, the weaker and more euphemistic these modal verbs are.

Based on Halliday (1994) value classification and Huddleston and Pullum (2002) semantic analysis of modal verbs, modal verbs in this paper are divided into four groups based on their values. There will be “can/may/could/might” for Value 1, “would” for Value 2, “shall/should” for Value 3, and “must” for Value 4 (Table 1).

**Table 1:** Value classification of English modal verbs.

Value	1	2	3	4
Modal verbs	Can, could, may, might	Would	Shall, should, will	Must

According to their values, Chinese modal verbs can also be divided into several groups. Xu (2018) identified twenty-four Chinese model verbs. The formality of GPCs, however, reduces the number of Chinese modal verbs found in the corpus to nineteen. Table 2 shows the categorization of these nineteen Chinese modal verbs according to Li (2018) and Xu (2018), as well as the values of their English counterparts.

**Table 2:** Chinese modal verbs values.

Value	1	2	3	4
Modal verbs	可以keyi, 大概dagai, 能够nenggou, 可能keneng, 也许yexu	希望xiwang, 愿yuan, 将要jiangyao	要yao, 应该yinggai, 得de, 想xiang, 如果ruguo, 应当yingdang	必须bixu, 必然biran, 肯定kending, 一定yiding,势必shibi

### Research questions

Studies on modal verbs tend to focus on written rather spoken aspects. Using computer learner corpora, Aijmer (2002) compared the range and frequency of some key modal words between native English and advanced English learners’ writing using a 52,000-word corpus. The findings show that L2 writers overused modal verbs, a tendency that may be both developmental and interlingual. Chinese speakers who use English as a foreign language may overuse verbs because Chinese is a verb-heavy language (Pease and Cheung, 2018). While Aijmer’s study reports the frequency of different modal verbs in both corpora, it fails to explain why the frequency varies from one modal verb to another. Studies that investigate modal verbs used in oral output tend to use corpora with limited sizes. Park (2019) examined the distribution of modal verbs in a corpus of about 55,000 spoken words and concluded that English learners from Korea produced fewer modal verbs than native speakers, contradicting Aijmer (2002) findings. Römer (2004) conducted a comparative analysis of modal verbs by using spoken British English corpus data and data from English as a foreign language (EFL) textbooks and found discrepancies between authentic English and English textbooks in terms of the use of modal verbs. The modals *will*, *can*, and *must* were overused in the textbooks, while *would*, *could*, *should*, and *might* were underused.

Frequently regarded as a challenging grammatical structure in English (Palmer, 2014), modal verbs pose one of the greatest challenges for EFL learners (Saeed, 2009; Bensaid, 2016; Cournane and Pérez-Leroux, 2020). The misuse of modal verbs by EFL learners could result in unintended pragmatic consequences in cross-language communication contexts (Hyland and Milton, 1997). Markkanen and Schroder (1997) stressed the importance for EFL leaners to become familiar with the use of modal verbs to avoid potential misunderstanding. Therefore, observing EFL speakers, including interpreters, is imperative for shedding light on the link between use of modal verbs in EFL and effective cross-cultural communication.

Most corpus-based investigations of modal verbs are found in the translation literature instead of interpreting. Kranich’s (2009) study of the translation of scientific texts from English to German revealed that translators tend to use higher-value epistemic modals by using a relatively large corpus of over 500,000 tokens; however, it only examined popular scientific texts. Zhao and Liang (2013) compared the English–Chinese translation of the epistemic verbs *may* and *might* between literary and non-literary texts, thus expanding the scope of study to literary translation. Wu (2019) explored the differences between four Chinese translations of Shakespeare’s *Measure for Measure* in terms of *can*, *may*, *must*, *shall*, and *will*. Pei and Li (2018) investigated the distribution of the semantic meanings and values of more than ten Chinese and English modal verbs in thirty-three Chinese civil–commercial legislative instruments and their English translations.

There are few studies on the interpreting of modal verbs. Warchał and Łyda (2009) were the first to study modal verbs in interpreting and collected eighteen student interpreters’ Polish–English and English–Polish consecutive interpretations and examined their transfer of epistemic modal markers. However, with a “translation failure” of about 21% (Polish–English) to 26% (English–Polish), it seems that this research’s primary focus was on L2 learners’ utilization of modal verbs and thus will not provide any insights into the interpreting of modal verbs.

Chinese scholars (Li and Hu, 2013; Li, 2018; Fu and Chen, 2019) have used the GPC corpus to study the shift of modal verbs identified in English interpretation. Their findings suggest that such a shift could be attributed to the need to follow the conventions in the target language. However, none of these studies explored the modal verb distribution in both the ST (source text) and TT (target text), nor did they address the difference in values between modal verbs within the comparable pair. This fails to help us to understand how modal verbs are interpreted in the GPCs. Most of the research has focused on the utilization of modal verbs of strong value and the function of low-value modal verbs in interpretation has been neglected. As one of the most important and difficult grammatical systems in English (Palmer, 2014), modal verbs indicate proficiency in English (Thomas, 1994; Römer, 2004).

This study addresses this research gap by examining the distribution and value of modal verbs in both the ST and TT for a more accurate description of Chinese–English interpreting in terms of modality. Drawing on a self-built corpus of GPC interpreting, this article attempts to conduct a comprehensive intertextual analysis and seeks possible explanations behind the statistics. To this end, this study attempts to answer the following two questions:

1. What are the emergence frequencies of modal verbs with different values in the comparable corpus?
2. What are the interpreting methods for modal verbs, and how they are distributed?

## Corpus and procedures

The corpus is comprised of twenty press conference sessions recorded during the Two Sessions of the National People’s Congress and the Chinese People’s Political Consultative Conference from 2003 to 2022 and delivered by two premiers of China, as well as five interpreters. The corpus consists of 309,737 tokens in total (128,960 in English and 180,777 in Chinese), and a total recording length of 2,541 min. Additionally, to understand if the use of modal verbs by interpreters was similar to that of native English speakers, we compared the figure in GPC interpretation with the original English GPC corpus created by Li and Hu (2013), which contains 223,728 tokens derived from thirty press conferences held between 1989 and 2011. According to Quirk et al. (1985), Biber et al. (1999), Mindt (2000), and Facchinetti et al. (2003), the central modal verbs (*can*, *could*, *may*, *might*, *must*, *shall*, *should*, *will*, and *would*) are the most typical and frequently used. These modal verbs will be discussed in this study, with an emphasis on their affirmative versions.

The frequency of modal verbs (nineteen in Chinese and nine in English) in the ST and TT in the corpus will first be reported. Then, the analysis characteristics of the use of modal verbs in Chinese–English interpreting of GPCs by comparing the frequency and value of Chinese modal verbs in the ST and their English counterparts in the TT will be presented. Finally, the Chinese into English interpretation of modal verbs will be categorized and analyzed.

## Analysis and results

### Distribution of modal verbs in ST and TT

Table 3 shows that the proportion of modal verbs in English interpretation is higher than that in Chinese STs. The English corpora contain more modal verb use than the Chinese corpora. Due to its paratactic nature, Chinese uses covert coherence to illustrate grammatical relationships in loosely structured, short, and simple sentences (Cheung, 2009a). In contrast, the English language emphasizes the logic and completeness of sentence structures by using modifiers. Thus, when interpreting Chinese into English, interpreters may have to make explicit in the English target language what is implicit in the Chinese source language (Cheung, 2007, Cheung, 2009a,b, 2016). This could account for the differences in the number of modal verbs between the Chinese and English corpora.

**Table 3:** Comparison of the total of modal verbs in ST and TT.

Corpora	Tokens	Modal verbs	Percentage
Chinese ST	180, 777	1,768	0.98
English TT	128, 960	2,658	2.06

Table 4 shows that the most frequently used Chinese modal verb is “要,” which has the approximate meaning of “should” in English. The second most frequent is “想,” meaning “will” in English.

**Table 4:** Distribution of Chinese modal verbs.

Chinese modal verb	Number	Percentage	Value
也许 yexu	3	0.17	1
大概dagai	11	0.62	1
可能keneng	108	6.11	1
能够nenggou	137	7.75	1
可以keyi	179	10.12	1
将要 jiangyao	3	0.17	2
希望xiwang	78	4.41	2
愿yuan	105	5.94	2
应当yingdang	16	0.90	3
得dei	115	6.50	3
如果ruguo	122	6.90	3
应该yinggai	124	7.01	3
想xiang	200	11.31	3
要yao	408	23.08	3
肯定 kending	3	0.17	4
势必 shibi	4	0.23	4
必然biran	7	0.40	4
一定yiding	57	3.22	4
必须bixu	88	4.98	4
Total	1768	100	N.A

Adopting from the typology of modality in Halliday (1994) and the scale of modal orientation in Li (2018), all modal verbs were assigned a numerical value from 1 to 4. Table 4 shows that the two most frequently used modal verbs in the Chinese are Value 3, accounting for 55.7% of all modal verbs. A total of 9% of modal verbs are found to be Value 4, which is the lowest of all four types. According to Perkins (1983), there are only sixteen modal verbs in English, while there are twenty-four in Chinese (Xu, 2018). Chinese speakers have more choices of modal verbs than English speakers, and this fact has an important effect on our corpora. The distribution of Chinese modal verbs is not as centralized as English. The figure may be indicative of the preference for more formal language in press conferences on the part of the Chinese premiers, as well as attempts at avoiding ambiguity and conveying toughness.

A total of 2,658 English modal verbs were identified from this corpus of 128,960 words, of which the “will” and “shall” verbs were manually differentiated into modal verbs and auxiliary verbs. Table 5 shows that the most frequently used modal verb is “will,” appearing 1,320 times among 128,960 tokens and making up nearly half of the modal verbs in the interpretation. The proportion of English modal verbs in the TT with the value of 3 increases, while the proportion of modal verbs with the value of 4 decreases, when compared to Chinese modal verbs in the ST. The modal verbs used most frequently after “will” are “can,” “would,” “must,” and “should.” Table 5 shows the frequency of each modal verb in English.

**Table 5:** Frequency of each English modal verb in TTs.

English modal verb	Number	Percentage	Value
might	14	0.53	1
could	61	2.29	1
may	124	4.67	1
can	337	12.68	1
would	266	10.01	2
shall	5	0.19	3
should	265	9.97	3
will	1,320	49.66	3
must	266	10.01	4
ALL	2,658	100	N.A

### Comparison of modal verbs in the English interpretation and original English GPC corpora

Table 6 shows the similarities and differences between how often modal verbs are used in English-interpreted Chinese GPCs compared to the native-English ones. Frequency in Table 6 refers to the how often a modal verb is used per thousand words of the entire corpus, while percentage refers to how frequently each modal verb is used per hundred modal verbs. A list of the modal verbs investigated by Li and Hu (2013, 28) appears in the Original English GPC column.

**Table 6:** Comparison of modal verbs in bilingual/English-speaking press conferences.

——	Interpreted English			Original English GPC		
Modal verb	Counts	Frequency*	Value	Counts	Frequency*	Value
can	337	2.61‰	1	712	3.18‰	1
could	61	0.47‰	1	236	1.05‰	1
may	124	0.96‰	1	106	0.47‰	1
might	14	0.11‰	1	125	0.56‰	1
would	266	2.06‰	2	925	4.13‰	2
shall	5	0.04‰	3	2	0.01‰	3
should	265	2.05‰	3	246	1.1‰	3
will	1,320	10.24‰	3	1,416	6.33‰	3
must	266	2.06‰	4	84	0.38‰	4
Total	2,658	20.06‰	N.A	3,852	17.22‰	N.A

*Every thousand words.

Table 6 shows that the overall frequency of modal verbs in English interpretation is 20.06‰, which is 16% higher than the English-speaking GPCs. However, the difference between the usages of each modal verb is even more noticeable.

On the one hand, in interpretation, low-value modal verbs are used less frequently. Modal verbs such as “can,” “could,” and “might” are rarer in the interpretation than in English-speaking GPCs. As Table 6 shows, “could” shows up only 0.47 times every 1,000 words in interpretations of Chinese GPCs, while it shows up much more regularly in English GPCs. This difference is also true of the usage of “might.” However, “may” is an exception to this rule. Although “may” is low-value, it is more frequently used in Chinese GPCs (0.96 times per 1,000 words) than in English-speaking ones (0.47 times per 1,000 words).

On the other hand, modal verbs of a medium or higher value are used much more frequently in the interpretation of Chinese GPCs than in English-speaking ones. As Table 6 shows, “must,” “shall,” and “should” are used more often in interpretation. The results show that the word “should” appears in English translations of Chinese GPCs nearly twice as often as it appears in English press conferences. In the former case, it occurs 2.05 times for every 1,000 words, while for the latter, it appears 1.1 times for every 1,000 words. The biggest gap in the usage of high-value modal verbs is “must.” The data show that “must” appears in the interpretation 2.06 times for every 1,000 words spoken, five times that of the English-speaking GPCs, suggesting that modality value tends to be intensified when interpreting from Chinese into English.

Table 7 shows the differences in the average of modal values among the corpora.

**Table 7:** Average value of modal values.

	ST	TT	Original English GPC
Average modal values	2.49	2.60	2.17

Table 7 shows that the TT has the highest (2.60) average modal value, followed by ST (2.49), while Original English GPC has the lowest (2.17) among the three. The difference between the TT and ST is 0.11. However, the difference between the TT and the Original English GPC is 0.43, or 19.382%. This difference seems to contradict the claim that the need to comply with the conventions of the target language leads to shifts in modal value when interpreting from Chinese into English (Li and Hu, 2013; Li, 2018; Fu and Chen, 2019). A negligible difference in the average of modal value between TT and Original English GPC may support that claim. However, a nearly 20% difference in average modal values between the TT and Original English GPC implies that there could be other reasons to account for an increase of modal values in TT.

### Interpreting modal verbs from Chinese into English

Interpreted modal verbs fall into five categories: deletion, amplification, direct translation, switching between affirmation and negation, and using modal verbs of different values. As Table 8 shows, only 39.69% of the modal verbs in the interpretation are directly derived from the ST without value change, while a similar percentage of modal verbs are additions in the TT that did not have a corresponding modal verb in the ST. Additionally, 20.62% of the modal verbs have their value changed in the process of interpreting, and fewer than 1% of the modal verbs are shifted between affirmative and negation. It is worth mentioning that 141 (7.98%) of the modal verbs used in Chinese are omitted in English.

**Table 8:** Categories of how modal verbs were interpreted into English.

	Counts in ST	Counts in TT	Percentage of TT
Omission	141	0	0%
Amplification	0	1,031	38.79%
Direct translation	1,055	1,055	39.69%
Switch between affirmative and negation	24	24	0.90%
Adjusting modal values	548	548	20.62%
Total	1768	2,658	100%

### Amplification

Amplification refers to the addition of a modal verb in the interpretation that is not found in the source language. As shown in Table 9, the interpretation contains a total of 2,658 modal verbs, of which 1,031 are the results of amplification, making up 38.79% of all the modal verbs. Table 9 also shows that high-value modal verbs such as “should,” “will,” and “must” take up the smallest percentage of amplification. Modal verbs that make up the highest proportion are “might” (78.57%), “shall” (80%), and “could” (55.1%).

**Table 9:** Amplification used on each modal verb.

Modal verb	Counts	Amplification	Percentage
will	1,320	480	36.36
can	337	152	45.10
would	266	141	53.01
must	266	56	21.05
should	265	103	38.87
may	124	51	41.13
could	61	33	54.10
might	14	11	78.57
shall	5	4	80.00
ALL	2,658	1,031	38.79

Two primary reasons may account for the use of amplification. First, when interpreting from Chinese to English, meanings that are implicit in Chinese need to be made explicit so that the interpretation is comprehensible to English speakers; hence, extra words are added (Cheung, 2009a,b), including modal verbs. The following examples illustrate the amplification of modal verbs in the target language.

#### Example 1a

##### Premier Wen

葬我于高山之上兮, 望我大陆 … 葬我于高山之上兮, 望我故乡。(2003).

##### GLOSS

Bury me on the highest mountaintop, staring at my mainland. Bury me on the highest mountaintop, looking afar my hometown.

##### Interpreter

Bury me on the highest mountaintop, so that I *can* get a sight of my mainland. Bury me on the highest mountaintop, so that I *can* get a glimpse of my hometown.

#### Example 1b

##### Premier Wen

诚实守信, 责权统一。(2004).

##### GLOSS

Good faith, trustworthiness, and responsibilities and rights in consistency.

##### Interpreter

The government *must* also be clean, honest, and honors its commitments. And there *must* be a combination of power and responsibility.

#### Example 1c

##### Premier wen

华山再高, 顶有过路。(2010).

##### GLOSS

Although Huashan Mountain is towering, the mountaintop is accessible.

##### Interpreter

No matter how high the mountain is, one *can* always ascend to its top.

In Example 1 a-c, the Chinese STs with four-character formulaic expressions. All three are null sentences that are grammatical in Chinese and comprehensible to Chinese speakers. However, the interpreter has to make the implicit subject and modal meaning explicit when interpreting into English so that the interpretation is grammatical and comprehensible in English. The hidden modal meaning in the source language should be amplified in the interpretation because of the differences between the two languages.

#### Example 2

##### Premier Li

至于说中日韩自贸区和RCEP哪一个先达成, 我想那要看我们各方所做的努力了。不管是哪一个协议能够先达成, 中方都乐见其成。(2019).

##### GLOSS

As to the China–Japan–ROK FTA or RCEP, which will be concluded first, I think that depends on how the parties concerned make efforts. And whichever agreement will come first, China welcome that.

##### Interpreter

As to which one will be concluded first, the China–Japan–ROK FTA or RCEP, I think that depends on efforts made by the parties concerned. And whichever will be concluded first, China would welcome that.

In Example 2, Premier Li stressed that as a strong advocate of free trade, China welcomes both, and there is no priority between the two. The word “would” not only emphasizes the Chinese government’s eagerness to promote China–Japan–ROK FTA and RCEP but also projects China’s inclusive attitude when conducting international affairs.

#### Example 3

##### Premier Wen

第二个阶段就是实行工业反哺农业, 城市支持农村的方式, 对农民多予、少取、放活。(2005).

##### GLOSS

In the second phase, we make industry nurture agriculture and cities support the countryside. For farmers, we give more, take less, and liberalize the countryside.

##### Interpreter

In the second phase, we should make industry nurture agriculture and cities support the countryside. We should give more to, take less from, and liberalize the countryside.

Rural development has always been a priority of the Chinese government. In Example 3, Premier Wen talks about how the government would promote reform and development of China’s rural areas, and he uses three two-character modifier-head structures to express his strong determination and concern about the rural areas. Modal verbs serve a variety of pragmatic functions, including marking evidentiality, possibility, likelihood, determination, strategic vagueness, and politeness (Markkanen and Schroder, 1997). So in order to achieve a similar pragmatic function in English, the word “should” is added to indicate the premier’s determination and confidence in rural development.

### Switch between affirmation and negation

There are twenty-four cases of switching modal verbs between affirmation and negation. Of the twenty-four conversions, twenty-one are changed from negative in Chinese to positive in English, probably to follow language conventions in English, as in the following example.

#### Example 4

##### Premier Wen

中国的主权和领土完整**不容**分割。(2006).

##### GLOSS

China’s sovereignty and territorial integrity brook no division.

##### Interpreter

China’s sovereignty and territorial integrity should always be intact.

This is Premier Wen’s reply to a question about the cross-strait relationship. Example 4 shows how the meanings of the source language are presented by using very different wordings in the target language. In this case, instead of saying what should not happen, the idea is formulated in a positive way—that is, what *should* happen.

### Adjusting modal values

Modal value also correlates with levels of politeness (Leech, 1987). Analyzing the difference, if any, between the ST and TT modal values may reveal the level of certainty between the two languages. Analyzing the changes in the value of each modal verb between the ST and TT may provide insights into the handling of modal verbs in settings characterized by cross-cultural and cross-linguistic communication. As Table 10 shows, 548 modal verbs in the TT experienced value changes in comparison to their Chinese counterparts. Of those, 285 are rendered into higher-value modal verbs in the TT, and 262 into lower-value ones. The value difference was calculated by comparing the value in the interpretation to the value in the original text. For example, if the value in the TT is 3, and the value in the ST is 1, the difference in value is 2. The value deviation of modal verbs from the ST to the TT was within the range of −2 to 2. There are no instances where the differences are either 3 or −3, suggesting that there are no cases in which there is an extreme difference in the modal value of the ST and TT. Table 10 shows that in most of the value changes, the value variation between the ST and TT is only 1.

**Table 10:** Modal verbs’ value change between ST and TT.

Value changes (TT’s value-ST’s value)	Counts
−3	0
−2	31
−1	231
1	257
2	28
3	0
Total	548

#### Example 5

##### Premier Li

这几年的实践可以证明, 中国经济“硬着陆”论**可以**休矣。(2017).

##### GLOSS

The past few years’ economic performance demonstrate that the predictions of China’s “hard landing” should be put to an end.

##### Interpreter

I hope this will put an end to any more predictions of a hard landing.

High-value modal verbs could be used to express strong attitudes. In Example 5, to illustrate the premier’s firm objection to the narrative of China’s looming economic hard landing, the value of the modal verb is adjusted from 1 in the ST to 3 in the TT. As such, the attitude in English is stronger than that in Chinese, with a difference of 2.

#### Example 6

##### Premier Wen

同时, 我也**必须**说明, 这是我们主动调控的结果。(2012).

##### GLOSS

At the same time, I must say that this is exactly what our proactive regulations have delivered.

##### Interpreter

At the same time, I would like to say that the economic slowdown in China is mainly the result of our proactive macro control.

Premier Wen is responding to a question on the slowdown of China’s economic growth in Example 6. In the source language, the modal verb “必须,” which means “must” in English, has a modal value of 4, indicating a very strong stance and sentiment. However, the word “would,” with a modal value of 3, was used in English, potentially projecting a softer and gentler image of China in English than in Chinese.

#### Example 7

##### Premier Wen

经济发展快了也不行, 经济生活长期处于紧张阶段, 难以为继。(2005).

##### GLOSS

Yet too fast economic growth rate will not work either because the economy life will face long-term stretch-out in an unsustainable situation.

##### Interpreter

Yet too fast economic growth rate will not do either because it may make the economy to stretched out for a long time in an unsustainable situation.

In Example 7, when responding to a question about the speed of economic growth, Premier Wen concentrates on the possible dangers of hasty economic development. Instead of using the Value 3 modal verb “will,” which has an equivalent modal value in English, the word “may,” which is a Value 1 modal verb, is used to increase the level of uncertainty in the prediction.

### Omission

Omission means no trace of the ST modal verb is found in the TT. Results show that 7.98% of the modal verbs in the ST were omitted. Chinese–English interpreting usually uses omission to eliminate redundant words to be idiomatic in English, as the following example shows.

#### Example 8

##### Premier Li

而且在此基础上, 由政府和居民共担, 购买大病保险, 建立了大病保险的机制, 这是**可以**缓解大病患者特别是困难群众负担的一个重要举措。(2019).

##### GLOSS

Moreover, on this basis, the government and residents jointly take the risk, purchase serious illness insurance, and establish a mechanism of serious illness insurance, this is an important measure that could alleviate the burden of patients with serious illness, especially the people with difficulties.

##### Interpreter

In addition, we have established the scheme for serious illness insurance with a cost-sharing formula between the government and individuals, an important measure to mitigate the burdens on patients with serious illnesses, especially needy patients.

In Example 8, Premier Li introduces a policy the Chinese government has taken to reduce patients’ financial burdens, but when he describes the purpose of this policy, he uses the modal verb “可以,” which has a low value of 1. The statement may be perceived as uncertain and less convincing with the presence of this low-value modal. Thus, the interpreter omits the “可以” included in the ST and places “重要举措” (important measure) at the beginning of the clause to signal the Chinese government’s commitment to improving social welfare.

## Conclusion

The study analyzed the frequency and features of modal verbs and how they are interpreted from Chinese into English in corpora with twenty GPCs from the Two Sessions. The findings only partly support the claim that the need to follow target language conventions leads to shifts in modal value during the process of interpreting Chinese into English (Li and Hu, 2013; Li, 2018; Fu and Chen, 2019). The difference in the average modal values between the original English corpus and the interpretation corpus suggests that other factors may also contribute to these shifts. One such factor may be varying signals between domestic and overseas listeners. The differences in modal values between the ST and TT indicate that the value shifts may be attributed to the need to convey semantic and pragmatic functions in the target language. The current study also categorized the different interpreted modal verbs, an area that has not been extensively studied, and found that only 40% of the modal verbs were rendered literally in the TT. Quite a few modal verbs in the TT were amplified, partly because of the need to make what is implicit in the ST explicit in the TT. Some modal verbs were omitted in the TT, but the intended pragmatic functions of these omitted modal verbs were retained by sentence restructuring in the TT. Other approaches, such as using euphemism and adverbs in the TT, may have similar functions but will be explored in the future.

At least two factors limit the generalizability of this study. First, all interpreters are not native English speakers and might have used modal verbs unidiomatically, despite being highly proficient in English. Second, the setting of the Two Sessions is formal and heavily scripted; data from settings that are less formal and unscripted may yield different results. Future studies should investigate the use of modal verbs in diverse settings and test related hypothesis with inferential statistics.

## Data availability statement

The original contributions presented in the study are included in the article/Supplementary material, further inquiries can be directed to the corresponding author.

## Author contributions

YZ performed data analysis and wrote the first draft of the manuscript. AC contributed to data-coding and interpretation of results. All authors contributed to the article and approved the submitted version.

## Funding

This paper is sponsored by the Fundamental Research Funds for the Central Universities HUST: 2016AC027.

## Conflict of interest

The authors declare that the research was conducted in the absence of any commercial or financial relationships that could be construed as a potential conflict of interest.

## Publisher’s note

All claims expressed in this article are solely those of the authors and do not necessarily represent those of their affiliated organizations, or those of the publisher, the editors and the reviewers. Any product that may be evaluated in this article, or claim that may be made by its manufacturer, is not guaranteed or endorsed by the publisher.

## References

[ref1] Aijmer K. (2002). “Modality in advanced Swedish learners' written interlanguage” in Computer Learner Corpora, Second Language Acquisition and Foreign Language Teaching. eds. Granger J. H. Petch-Tyson S. (Amsterdam: John Benjamin Publishing Company), 55–76. doi: 10.1075/lllt.6.07aij

[ref2] Bensaid M. (2016). Arab ESL learners and modals. Arab World Eng. J. 6, 90–97. doi: 10.2139/ssrn.2843929

[ref3] Biber D. Johansson S. Leech G. Conrad S. Finegan E. Hirst G. (1999). The Longman grammar of spoken and written English. TESOL Q. 35, 1265–1269. doi: 10.1016/S0378-2166(03)00029-8

[ref4] Cheung A. K. F. (2001). Code-mixing and simultaneous interpretation training. Interpreters’ Newsl. 11, 57–62.

[ref5] Cheung A. K. F. (2003). “Does accent matter? The impact of accent in simultaneous interpretation into mandarin and Cantonese on perceived performance quality and listener satisfaction level,” in Evaluación de la Calidad en Interpretación de Conferencia Investigación. eds. Aís A. C. Sanchez M. F. Gile D. (Granada: Interlingua) 85–96.

[ref6] Cheung A. K. F. (2007). The effectiveness of summary training in consecutive interpreting (CI) delivery. Forum 5, 1–23. doi: 10.1075/forum.5.2.01che

[ref7] Cheung A. K. F. (2008). Simultaneous interpreting of numbers: an experimental study. Forum 6, 23–38. doi: 10.1075/forum.6.2.02kfc

[ref8] Cheung A. K. F. (2009a). 中英交替传译之显化现象:案例分析 (Explicitation in consecutive interpreting from Chinese into English: a case study). Chinese Translators J. 5, 77–81.

[ref9] Cheung A. K. F. (2009b). Numbers in simultaneous interpreting: an experimental study. Forum 7, 61–88. doi: 10.1075/forum.7.2.03che

[ref10] Cheung A. K. F. (2012a). The use of reported speech by court interpreters in Hong Kong. Interpreting 14, 73–91. doi: 10.1075/intp.14.1.04che

[ref11] Cheung A. K. F. (2012b). Omission in simultaneous interpreting: word order differences. Forum 10, 19–33. doi: 10.1075/forum.10.2.02che

[ref12] Cheung A. K. F. (2013). Non-native accents and simultaneous interpreting quality perceptions. Interpreting 15, 25–47. doi: 10.1075/intp.15.1.02che

[ref13] Cheung A. K. F. (2014a). Anglicized numerical denominations as a coping tactic for simultaneous interpreting from English into mandarin Chinese: an experimental study. Forum 12, 1–22. doi: 10.1075/forum.12.1.01che

[ref14] Cheung A. K. F. (2014b). The use of reported speech and the perceived neutrality of court interpreters. Interpreting 16, 191–208. doi: 10.1075/intp.16.2.03che

[ref15] Cheung A. K. F. (2015). Scapegoating the interpreter for listeners’ dissatisfaction with their level of understanding: an experimental study. Interpreting 17, 46–63. doi: 10.1075/intp.17.1.03che

[ref16] Cheung A. K. F. (2016). Paraphrasing exercises and training for Chinese to English consecutive interpreting. Forum 14, 1–18. doi: 10.1075/forum.14.1.01che

[ref17] Cheung A. K. F. (2017). Non-renditions in court interpreting: a corpus-based study. Babel 63, 174–199. doi: 10.1075/babel.63.2.02che

[ref18] Cheung A. K. F. (2018). Non-renditions and the court interpreter’s perceived impartiality: a role-play study. Interpreting 20, 232–258. doi: 10.1075/intp.00011.che

[ref19] Cheung A. K. F. (2019). “The hidden curriculum revealed in study trip reflective essays,” in The Evolving Curriculum in Interpreter and Translator Education: Stakeholder Perspectives and Voices. eds. Sawyer D. B. Austermühl F. Raído V. E. (Philadelphia: John Benjamins Publishing Company), 393–408.

[ref20] Cheung A. K. (2020). Interpreters’ perceived characteristics and perception of quality in interpreting. Interpreting 22, 35–55. doi: 10.1075/intp.00033.che

[ref21] Cheung A. K. F. (2022). Listeners’ perception of the quality of simultaneous interpreting and perceived dependence on simultaneous interpreting. Interpreting 24, 38–58. doi: 10.1075/intp.00070.che

[ref22] Cournane A. Pérez-Leroux A. (2020). Leaving obligations behind: epistemic incrementation in preschool English. Lang. Learning. Dev. 16, 270–291. doi: 10.1080/15475441.2020.1738233

[ref23] Facchinetti R. Krug M. Palmer F. (2003). Modality in Contemporary English. Berlin: Mouton De Gruyter.

[ref24] Fu R. Chen J. (2019). Negotiating interpersonal relations in Chinese-English diplomatic interpreting: Explicitation of modality as a case in point. Interpreting 21, 12–35. doi: 10.1075/intp.00018.fu

[ref25] Halliday M. A. K. (1994). An Introduction to Functional Grammar. London: E. Arnold.

[ref26] Huddleston R. (1976). Some theoretical issues in the description of the English verb. Lingua 40, 331–383. doi: 10.1016/0024-3841(76)90084-X

[ref27] Huddleston R. Pullum G. (2002). The Cambridge Grammar of the English Language. Cambridge: Cambridge University Press.

[ref28] Hyland K. Milton J. (1997). Qualification and certainty in L1 and L2 students’ writing. J. Second Lang. Writing 6, 183–205. doi: 10.1016/S1060-3743(97)90033-3

[ref29] Kranich S. (2009). Epistemic modality in English popular scientific texts and their German translations. Trans. Kom. 2, 26–41.

[ref30] Leech G. (1987). Meaning and the English Verb. 2nd Edn. London: Longman.

[ref31] Li X. (2018). Mediation through modality shifts in Chinese-English government press conference interpreting. Babel 64, 269–293. doi: 10.1075/babel.00036.li

[ref32] Li R. Cheung A. K. F. Liu K. (2022). A corpus-based investigation of extra-textual, connective, and emphasizing additions in English-Chinese conference interpreting. Front. Psychol. 13:844735. doi: 10.3389/fpsyg.2022.847735 PMC9190950 35707653

[ref33] Li X. Hu K. B. (2013). 基于语料库的记者招待会汉英口译中情态动词的应用研究 [a corpus-based study of modal verbs in Chinese-English government press conference interpretation]. Technol. Enhanced Foreign Lang. Educ. 151, 26–32. doi: 10.3969/j.issn.1001-5795.2013.03.005

[ref34] Liu J. (1960). On auxiliary verbs. Stud. Chinese Lang. 1, 1–4.

[ref35] Lyons J. (1977). Semantics. Volume 2. Cambridge: Cambridge University Press.

[ref36] Ma X. Cheung A. K. F. (2020). Language interference in English-Chinese simultaneous interpreting with and without text. Babel 66, 434–456. doi: 10.1075/babel.00168.che

[ref37] Markkanen R. Schroder H. (1997). “Hedging: a challenge for pragmatics and discourse analysis: approaches to the analysis of a pragmatic phenomenon in academic texts,” in Hedging and Discourse: Approaches to the Analysis of a Pragmatic Phenomenon in Academic Texts. eds. Markkanen R. Schroder H. (Berlin: Mouton De Gruyter), 3–20.

[ref38] Mindt D. (2000). An Empirical Grammar of the English Verb System. Berlin: Cornelsen.

[ref39] Palmer F. R. (2014). Modality and the English Modals, 2nd Edn. London: Longman.

[ref40] Park S. (2019). The use of make in a Korean learner spoken corpus. Mon. Stud. Eng. Lang. Lit. 63, 49–68. doi: 10.17754/MESK.63.3.49

[ref41] Pease A. Cheung A. K. F. (2018). Toward a Semantic Concordancer. In Proceedings of the 9th Global WordNet Conference, 97–104.

[ref42] Pease A. Pease J. C. Cheung A. K. (2018). Formal ontology for discourse analysis of a corpus of court interpreting. Babel 64, 594–618. doi: 10.1075/babel.00054.pea

[ref43] Pei J. Li J. (2018). A corpus-based investigation of modal verbs in Chinese civil-commercial legislation and its English versions. Int. J. Legal Discourse 3, 77–102. doi: 10.1515/ijld-2018-2003

[ref44] Peng L. Z. (2007). Modern Chinese Modality Study. Beijing: China Social Sciences Press.

[ref45] Perkins M. (1983). Modal Expressions in English. London: F. Pinter.

[ref46] Quirk R. Greenbaum S. Leech G. Svartvik J. (1985). A Comprehensive Grammar of the English Language. London: Longman.

[ref47] Römer U. (2004). “Comparing real and ideal language learner input: the use of an EFL textbook corpus in corpus linguistics and language teaching,” in Corpora and Language Learners. eds. Aston G. Bernardini S. Stewart D. (Philadelphia: John Benjamins Publishing Company), 151–168.

[ref48] Saeed A. (2009). Arab EFL learners’ acquisition of modals. Res. Lang. 7, 75–98. doi: 10.2478/v10015-009-0006-5

[ref49] Setton R. (2011). “Corpus-based interpreting studies (CIS): overview and prospects,” in Corpus-Based Translation Studies: Research and Applications. eds. Kruger A. Wallmach K. Munday J. (London: Bloomsbury Publishing PLC), 33–75.

[ref50] Sinclair J. M. (1990). Collins Cobuild English Grammar. London: Harpercollins.

[ref51] Song S. Cheung A. K. F. (2019). Disfluency in relay and non-relay simultaneous interpreting: an initial exploration. Forum 17, 1–19. doi: 10.1075/forum.18016.che

[ref52] Thomas M. (1994). Assessment of L2 proficiency in second language acquisition research. Lang. Learning. 44, 307–336. doi: 10.1111/j.1467-1770.1994.tb01104.x

[ref53] Tsang C. (1981). A Semantic Study of Modal Auxiliary Verbs in Chinese. Ph.D. Thesis. California: Stanford University

[ref54] Warchał K. Łyda A. (2009). “Epistemic modality markers in polish-English and English-polish consecutive interpreting: modal values and categories,” in W Kręgu Teorii: Studia Językoznawcze Dedykowane Profesorowi Kazimierzowi Polańskiemu in Memoriam. eds. Fontański H. Molencki R. Wolińska O. (Katowice: Uniwersytet Śląski), 247–265.

[ref55] Westney P. (1995). Modals and Periphrastics in English: An Investigation into the Semantic Correspondence Between Certain English Modal Verbs and Their Periphrastic Equivalents. Tfibmgen: Max Niemeyer Verlag.

[ref56] Wu B. (2019). Chinese translation of modal verb shall in Shakespeare’s measure for measure. Int. J. Ling. 11:53. doi: 10.5296/ijl.v11i5.15589

[ref57] Wu B. Cheung A. K. F. Xing J. (2021). Learning Chinese political formulaic phraseology from a self-built bilingual United Nations security council corpus: a pilot study. Babel 67, 500–521. doi: 10.1075/babel.00233.wu

[ref58] Xu Y. (2018). 机构新闻翻译情境下情态选择的编译改动与意识形态的转换. [the translation and change of modality and transformations in ideology in the context of institutional news translation] foreign Lang. Edu. 39, 93–97. doi: 10.16362/j.cnki.cn61-1023/h.2018.03.019

[ref59] Zhao Q. R. Liang M. C. (2013). 认识型情态动词may和might汉译强度变化研究 [The modal strength of epistemic verbs *may* and *might* in English-Chinese translation]. Shandong Foreign Lang. Teach. 157, 96–99. doi: 10.3969/j.issn.1002-2643.2013.06.017

[ref60] Zheng T. G. (2001). “Semantic property and syntactic property of auxiliary” in Chinese Language and Culture Study (Series 8). eds. Wenqing X. Hui S. (Beijing: Intellectual Property Publishing House), 60–73.

[ref61] Zhu Y. (1996). “Modality and modulation in Chinese,” in Meaning and Form: Systemic Functional Interpretations. eds. Berry M. Butler C. Fawcett R. Huang G. (Norwood, NJ: Ablex Publishing Corporation), 183–209.

